# Supplementary material for: Resolving the full spectrum of human genome variation using Linked-Reads
Source: Genome Res. 2019 Apr;29(4):635–45. doi: 10.1101/gr.234443.118 (PMC6442396; doi:10.1101/gr.234443.118)
Supplement: Supplemental Material [file supp_gr.234443.118_Supplemental_longranger-2.2.zip › longranger-2.2.tar.bz2!/lr2.2-public/lib/stan/cmdstan/stan/doc/api/html/fwd_2functions_2log10_8hpp_source.html]

Stan: src/stan/agrad/fwd/functions/log10.hpp Source File


|  |  |
| --- | --- |
|  | Stan  2.5.0  probability, sampling & optimization |


- Main Page
- Related Pages
- Namespaces
- Classes
- Files

- File List
- File Members

All Classes Namespaces Files Functions Variables Typedefs Enumerations Enumerator Friends Macros Pages

- src
- stan
- agrad
- fwd
- functions

log10.hpp

Go to the documentation of this file.

1 #ifndef STAN\_\_AGRAD\_\_FWD\_\_FUNCTIONS\_\_LOG10\_HPP

2 #define STAN\_\_AGRAD\_\_FWD\_\_FUNCTIONS\_\_LOG10\_HPP

3

4 #include <stan/agrad/fwd/fvar.hpp>

5 #include <stan/meta/traits.hpp>

6 #include <stan/math/constants.hpp>

7

8 namespace stan {

9

10  namespace agrad {

11

12  template <typename T>

13  inline

14  fvar<T>

15  log10(const fvar<T>& x) {

16  using std::log;

17  using std::log10;

18  using stan::math::NOT\_A\_NUMBER;

19  if(x.val\_ < 0.0)

20  return fvar<T>(NOT\_A\_NUMBER, NOT\_A\_NUMBER);

21  else

22  return fvar<T>(log10(x.val\_), x.d\_ / (x.val\_ \* stan::math::LOG\_10));

23  }

24  }

25 }

26 #endif

stan::agrad::log10

var log10(const var &a)

Return the base 10 log of the specified variable (cmath).

**Definition:** log10.hpp:55

stan::math::NOT\_A\_NUMBER

const double NOT\_A\_NUMBER

(Quiet) not-a-number value.

**Definition:** constants.hpp:53

stan::agrad::fvar

**Definition:** fvar.hpp:13

stan::math::LOG\_10

const double LOG\_10

The natural logarithm of 10, .

**Definition:** constants.hpp:38

traits.hpp

fvar.hpp

stan::agrad::fvar::d\_

T d\_

**Definition:** fvar.hpp:16

constants.hpp

stan::agrad::log

fvar< T > log(const fvar< T > &x)

**Definition:** log.hpp:15

stan::agrad::fvar::val\_

T val\_

**Definition:** fvar.hpp:15

stan::agrad::log10

fvar< T > log10(const fvar< T > &x)

**Definition:** log10.hpp:15


---

[ Stan Home Page ]

*© 2011–2014,
Stan Development Team.*
